# Computerized Clinical Decision Support System for Emergency Department–Initiated Buprenorphine for Opioid Use Disorder: User-Centered Design

**DOI:** 10.2196/13121

**Published:** 2019-02-27

**Authors:** Jessica M Ray, Osama M Ahmed, Yauheni Solad, Matthew Maleska, Shara Martel, Molly M Jeffery, Timothy F Platts-Mills, Erik P Hess, Gail D’Onofrio, Edward R Melnick

**Affiliations:** 1 Yale School of Medicine New Haven, CT United States; 2 Yale New Haven Health New Haven, CT United States; 3 The Patient Revolution New Haven, CT United States; 4 Mayo Clinic Rochester, MN United States; 5 University of North Carolina School of Medicine Chapel Hill, NC United States; 6 University of Alabama at Birmingham School of Medicine Birmingham, AL United States

**Keywords:** user-centered design, decision support systems, clinical, opioid-related disorders, opiate substitution treatment, health information technology

## Abstract

**Background:**

Emergency departments (EDs) frequently care for individuals with opioid use disorder (OUD). Buprenorphine (BUP) is an effective treatment option for patients with OUD that can safely be initiated in the ED. At present, BUP is rarely initiated as a part of routine ED care. Clinical decision support (CDS) could accelerate adoption of ED-initiated BUP into routine emergency care.

**Objective:**

This study aimed to design and formatively evaluate a user-centered decision support tool for ED initiation of BUP for patients with OUD.

**Methods:**

User-centered design with iterative prototype development was used. Initial observations and interviews identified workflows and information needs. The design team and key stakeholders reviewed prototype designs to ensure accuracy. A total of 5 prototypes were evaluated and iteratively refined based on input from 26 attending and resident physicians.

**Results:**

Early feedback identified concerns with the initial CDS design: an alert with several screens. The timing of the alert led to quick dismissal without using the tool. User feedback on subsequent iterations informed the development of a flexible tool to support clinicians with varied levels of experience with the intervention by providing both one-click options for direct activation of care pathways and user-activated support for critical decision points. The final design resolved challenging navigation issues through targeted placement, color, and design of the decision support modules and care pathways. In final testing, users expressed that the tool could be easily learned without training and was reasonable for use during routine emergency care.

**Conclusions:**

A user-centered design process helped designers to better understand users’ needs for a Web-based clinical decision tool to support ED initiation of BUP for OUD. The process identified varying needs across user experience and familiarity with the protocol, leading to a flexible design supporting both direct care pathways and user-initiated decision support.

## Introduction

### Background

Opioid use disorder (OUD) is an escalating public health crisis that has impacted all regions of the United States and represents a substantial portion of emergency department (ED) visits each year. An estimated 2.1 million people in the United States have OUD [1] and 275 million people have OUD worldwide [2]. More than 33,000 opioid-related deaths occur annually in the United States and 118,000 opioid-related deaths occur annually worldwide [3]. From 2016 to 2017, EDs experienced a 30% increase in visits for opioid overdose [4]. As the primary source of care for many people with OUD, the ED offers an important opportunity to engage patients receiving care for acute and comorbid conditions related to opioid use.

Buprenorphine (BUP), a partial opioid agonist often combined with an opioid antagonist, is a proven effective treatment for OUD that decreases mortality, withdrawal symptoms, craving, and opioid use [5-7]. Initiating BUP in the ED doubles the rate of addiction treatment engagement in ED patients with OUD [8]. However, ED-initiated BUP has not yet been adopted in most hospitals [9,10]. This delay in adoption of evidence-based practice is not unique—on average, it takes 17 years from discovery to the adoption of evidence-based practices into routine care [11,12].

Clinical decision support (CDS), computerized systems that offer patient-specific assessments or recommendations to clinicians, represents one approach to facilitating and accelerating the implementation process [13,14]. A 2011 review of randomized controlled trials investigating CDS guidance for drug therapy showed that CDS improved care in 64% (37/59) of studies [15]. In a broad review of CDS designed to address a range of care processes, meta-analysis favored the use of CDS for supporting clinician treatment orders (odds ratio 1.57, 95% CI 1.35-1.82). Large studies have shown CDS implementation in the ED to have supported the adoption of evidence-based practices for computed tomography imaging use [16,17].

However, CDS faces its own challenges, including unintended consequences such as alert fatigue and increased cognitive load [18-22]. CDS design principles support careful consideration of the sociotechnical environment and delivery of the right information, to the right person, in the right format, and at the right time in clinical workflow to optimize medical decision making [23-26].

Across the fields of technology and human-computer interaction, building usable systems has been found to be essential to improve efficiency and reduce errors [27]. International Organization for Standardization standards for user-centered design outline the process by which technological design can incorporate context and organizational requirements to produce and evaluate solutions [28]. The engagement of end users (the people who will be using the technology) throughout the process is critical to anticipate and avoid pitfalls of new information technology such as increased cognitive load and lack of user engagement [26]. Specifically, pragmatic approaches to usability evaluation are necessary to rapidly design, iterate, and test health care information systems [29].

### Objectives

Our objective was to develop a pragmatic, user-centered CDS for ED-initiated BUP and referral to treatment for patients with OUD. The user-centered design process for the development of this tool is described here. We developed this CDS specifically for the purposes of a planned multisystem pragmatic trial to study the effectiveness of user-centered CDS on adoption rates of ED-initiated BUP [30].

## Methods

### Clinical Context and Population

From March to July 2018, we utilized a multiphase, user-centered design methodology for the formative design, development, and evaluation of the EMergency department-initiated Buprenorphine for opioid usE Disorder (EMBED) CDS intervention. Primary phases in this method included (1) needs assessment, (2) initial prototype design, (3) iterative design feedback, and (4) final prototype testing. Formative feedback sessions were approved by our institution’s institutional review board; given the minimal risk of the study and a protocol that did not involve the collection of participants’ private information, all participants gave verbal consent for participation.

Eligible participants included ED clinicians and key stakeholders (including administrative and information technology leaders and ED addiction counselors) from an urban academic level I trauma center with 103,000 patient visits per year. Recruitment for user feedback sessions focused specifically on attending physicians and residents in the second, third, or fourth year of postgraduate medical training. During a 4-month period from March to June 2018, a total of 26 unique participants offered feedback during iterative design, including 14 through informal sessions and 12 through formal user feedback sessions. In addition, 6 participants offered feedback on multiple versions of the design. Informal sessions were conducted in the ED or private administrative offices and lasted 10 to 30 min. Formal sessions were conducted in the Yale Center for Medical Simulation and were approximately 45 min in length. Formal user design sessions were conducted in parallel with both attending and resident physicians by a human factors researcher (JR).

### Pragmatic Approach

Given our goal to rapidly increase adoption rates of ED-initiated BUP for a subsequent pragmatic trial, we elected to take a pragmatic approach to formative usability evaluation, as described by Mann et al [29]. This approach included rapid iterative design and testing cycles to provide user feedback and input on prototype design iterations. All sessions of user testing included direct observation, think aloud, and observational note-taking. In addition, notes were reviewed with each participant at the end of each session to ensure completeness. Pragmatic data analysis was performed by the design team during weekly meetings to debrief and summarize findings and to determine the design, functionality, and interface changes to make based on these findings. Termination was based on consensus, cost, and time constraints, as opposed to thematic saturation [29]. To minimize the assessment burden, we did not capture demographic data such as age, gender, race, or ethnicity (other than professional role) for the participants in the study [31].

#### Phase 1: Needs Assessment

The initial phase of design consisted of a focused discussion with key content and context experts as well as 3 ethnographic observation sessions of 2 to 5 hours in length. In the first 2 observations, the lead designer (MM) shadowed attending physicians in the ED. The third observation period focused on the processes of registration and the administration of patient flow through the waiting room and ED. Five 1-hour, individual interviews were then conducted with an ED drug and alcohol program counselor, a drug and alcohol treatment coordinator, an attending physician, and a resident. Interviews captured additional detail on workflow, roles, and user information needs.

#### Phase 2: Initial Prototype Design

After identifying potential users and their information needs, an initial low-fidelity prototype was designed. This prototype focused on key components necessary for implementing the ED-initiated BUP protocol, including modules to evaluate patients for OUD based on the Diagnostic and Statistical Manual of Mental Disorders, 5th Edition (DSM-5) criteria [32] and for opioid withdrawal severity using the Clinical Opioid Withdrawal Scale (COWS); the protocol for initiating BUP in the ED; and the steps necessary for referring patients for continued medication for OUD [33,34]. The initial prototype design was then reviewed by the design team as well as a subject matter expert on ED management of substance use disorder (GD) and a targeted sample of attending physicians and administrative leaders from the department. The goal of the initial prototype design phase was to establish the components necessary for the decision support tool and workflow. Questions identified during the initial review were addressed at this stage of design before moving forward to iterative design feedback sessions.

#### Phase 3: Iterative Design Feedback and Prototype Revision

With the initial static design complete, an interactive prototype was built in InVision (InVision, New York, NY). This prototype provided users with an interactive navigation and functionality experience. Feedback was gathered both through informal review and through formal user design sessions. Informal review included the distribution of electronic or print versions of all screens in the design to both attending and resident physicians. After verbal consent was obtained, each participant was oriented to the session format and read a case (Multimedia Appendix 1) of a patient presenting to the ED for treatment following an opioid overdose. Users were then given an electronic version of the CDS and asked to talk through how they would proceed. If participants did not initially mention the use of the tool, they were prompted to think about how and when they would expect to access the tool in this patient encounter. Participants were asked to think aloud describing how they expected to interact with the tool and were prompted for their initial reactions to it [35]. At the conclusion of each session, participants were asked to provide their overall impression of the tool’s content and format as well as suggestions to make the tool easier to use and to increase the likelihood of incorporating it into their practice. All data were entered in a design log identifying the user need, recommendation, and changes resulting from those recommendations. Recommendations were reviewed by the design team weekly to determine how they should inform design revisions. After each iteration, additional feedback sessions were conducted to gather additional data and further refine design.

#### Phase 4: Final Prototype Testing

Final testing of the interactive InVision prototype consisted of formal user feedback sessions that proceeded until the design team reached a consensus that the prototype would exceed all users’ needs 80% of the time based on the 80/20 rule [36]. These sessions followed the format of the formal iterative design feedback sessions (detailed above in Phase 3). Participants included both resident and attending physicians with a wide range of experience with the ED-initiated BUP. Sampling was deliberate to include both participants from earlier iteration sessions as well as new participants naïve to the user design.

## Results

### Phase 1 (Needs Assessments) and Phase 2 (Initial Prototype Design)

Overall, 4 key topics for design were identified in Phase 1 (Table 1). These initial areas of concentration included appropriate patient identification, defining potential users of the decision support tool, avoiding workflow disruptions, CDS steps, and supporting user understanding of the treatment process. Attending physicians were expected to be the target system users, yet early observations and feedback suggested parts of the decision process might be completed by other members of the care team, such as medical students, residents, or nurses. This broadened view of system users, with varying clinical roles and experience, became an ongoing design challenge driving decisions of how and when to present support.

**Figure 1:**
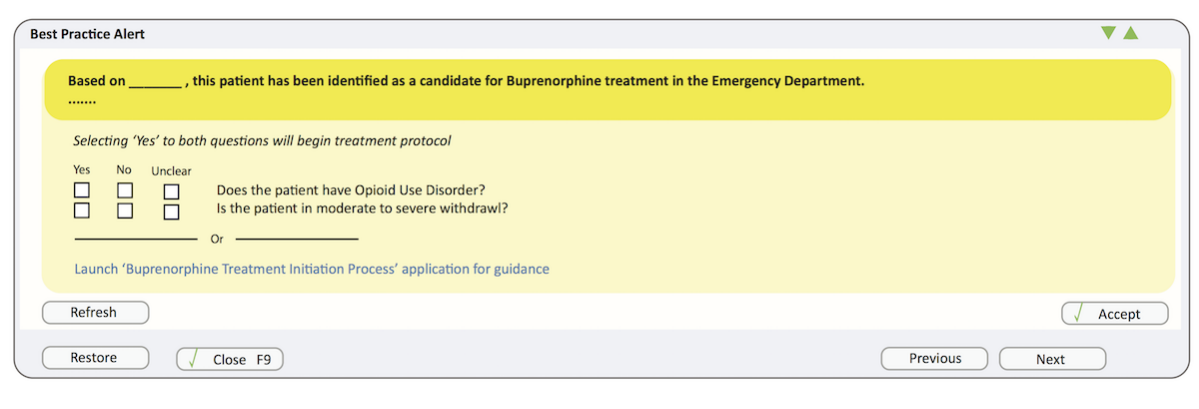
Initial prototype user interface mockup as Epic best practice alert.

Activation of CDS tools was an early feedback topic. The initial design was an Epic (Epic Systems, Verona, WI) best practice alert (BPA; Figure 1) triggering a pop-up window when a patient was identified as potentially having OUD. However, users disliked the pop-up alert format as it could easily be dismissed if triggered at the wrong time in the clinical workflow, potentially causing a missed opportunity to support the intervention.

A second area of concern for users (throughout all iterations) was avoiding workflow disruptions; they preferred that the tool take no longer than 2 to 5 min to use. Similarly, users highlighted the need for system flexibility to accommodate for the user’s experience level by allowing for decision support as needed as well as a direct care pathway selection with less support for more experienced clinicians.

**Table 1:** Needs assessment at baseline and ethnographic observation results.

Needs/topics	How they were expressed
Appropriate patient identification	Is it possible to have nurses identify patients with OUD^a^?
	Need to properly explain COWS^b^ to patients, who may understand it as “dope sick”
	Can discharge instructions for opioid abuse be a trigger to activate CDS^c^?
	There needs to be advanced search terms to trigger the CDS system—BPAs^d^ should not be the common denominator for analysis
Avoiding workflow disruptions	Avoid BPAs. They are intrusive and are rarely acted upon
	Sometimes, physicians do leave electronic health record to access MDCalc or clinical resources websites
	Attending physicians usually do not have time for decision support. Better to tailor this toward residents and nurses
	Entire intervention should take 2-5 mins to increase adoption
Streamlining CDS steps	Integrate COWS into the H & P^e^ template, with integrated decision support and order sets to determine the need for BUP^f^
	If a user is initiated for OUD diagnosis, then workflow should be streamlined and skip through the diagnostic criteria for OUD and go straight to treatment decision support
Understanding treatment process	Should patients be given a 4 mg or an 8 mg dosage?
	Need to have a short SBIRT^g^ included in CDS to assess patient willingness to begin treatment
	This is not the responsibility of our department but rather the substance abuse program
	Patients are rarely in the right range of withdrawal to prescribe BUP. Need to have a system to allow them to return at an appropriate time to the ED^h^
	Some patients may have a preference for suboxone versus methadone
	Some providers may have completed the waiver process but may not yet be recognized for it
	Should we have patients return to the ED for follow-up post BUP administration, using the 72-hour rule?

^a^OUD: opioid use disorder.

^b^COWS: Clinical Opioid Withdrawal Scale.

^c^CDS: clinical decision support.

^d^BPA: best practice alert.

^e^H & P: history and physical.

^f^BUP: buprenorphine.

^g^SBIRT: Screening, Brief Intervention, and Referral to Treatment.

^h^ED: emergency department.

**Figure 2:**
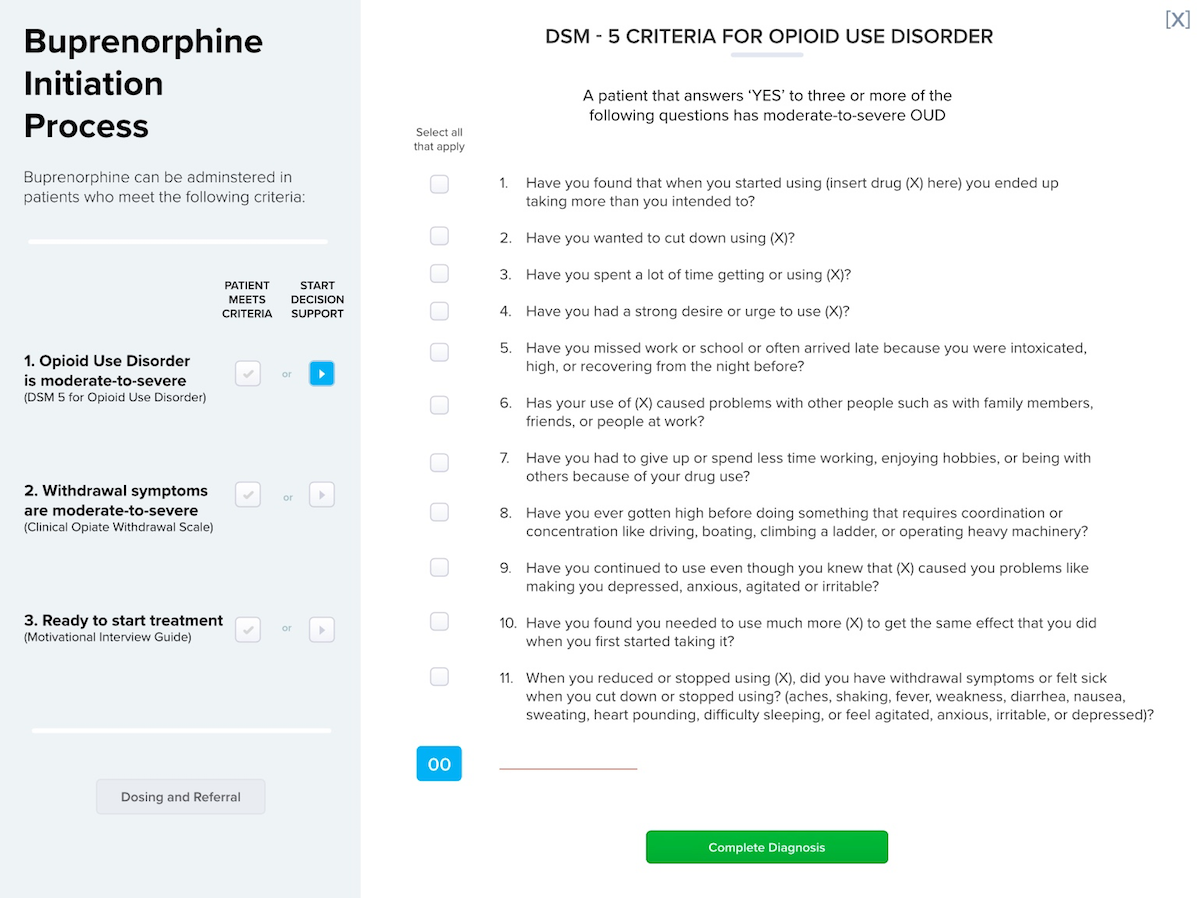
Second prototype user interface with optional decision support. DSM: Diagnostic and Statistical Manual for Mental Disorders; OUD: opioid use disorder.

These needs informed the development of the first prototype that incorporated existing paper forms into 1 process. This first iteration presented step-by-step guidance through 6 sequential screens (Figure 1 includes the first slide): introductory BPA, DSM checklist for diagnosing OUD, COWS withdrawal assessment, motivational interview prompts, treatment options, and a referral form. Each step was delegated to a single screen to emphasize the discrete steps in a streamlined workflow.

### Phase 3 (Iterative Design)

We created 5 major prototypes based on ongoing feedback. Multimedia Appendix 2 documents feedback received from each version and how it was incorporated into the subsequent revision. Across all prototypes, feedback focused on 4 thematic needs: design changes, navigation, workflow integration, and treatment process.

Feedback on the initial prototype (described above) focused on streamlining the prototype. This feedback was used to inform the second prototype (Figure 2) with the goal of a user-initiated CDS (instead of a BPA trigger) that could be embedded within the electronic health record (EHR) and further streamline the information in the individual steps.

Users found that this second iteration still had too many steps and too much text. They expressed difficulty in locating the decision support elements. Specific suggestions included consolidating steps with more clarity in regard to navigating the treatment options by including a progress bar.

**Figure 3:**
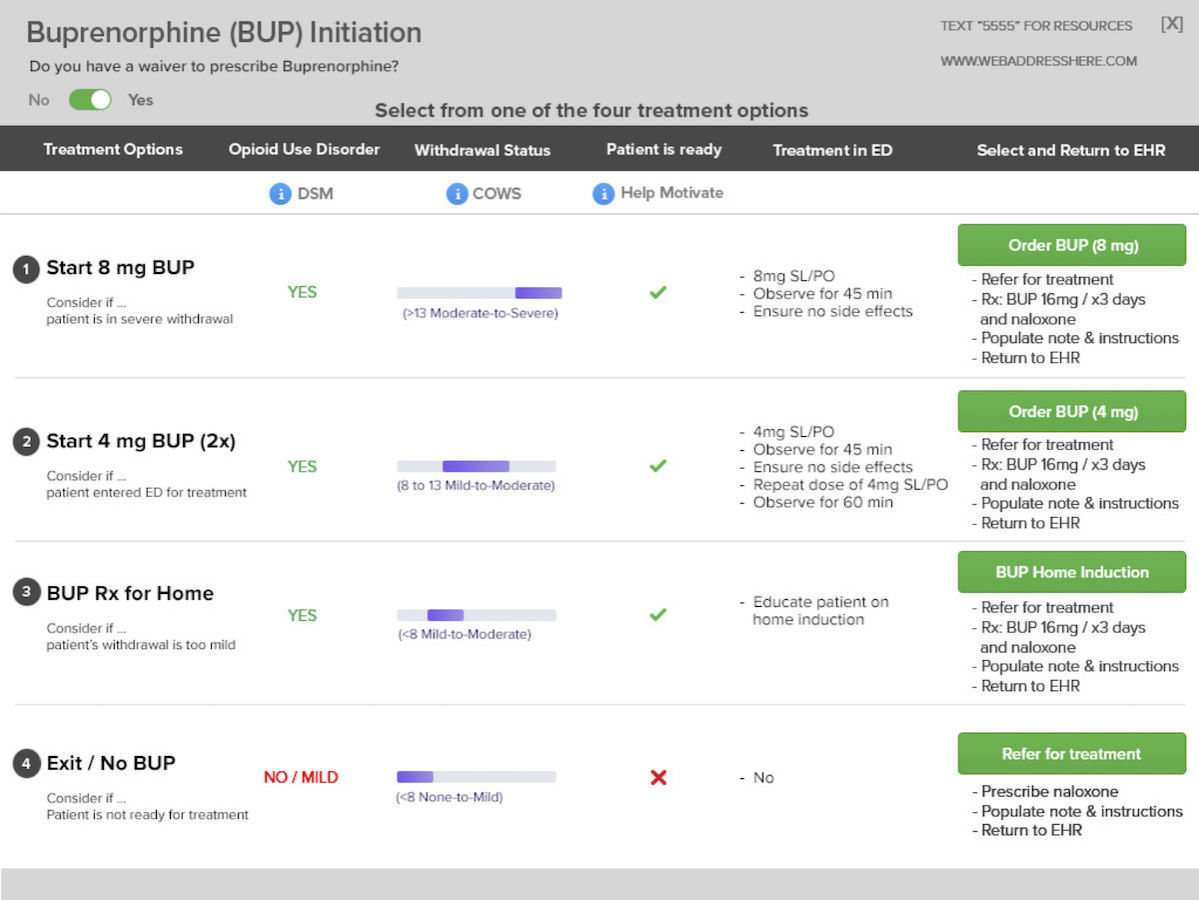
Third prototype user interface single-click care pathways. BUP: buprenorphine; COWS: Clinical Opioid Withdrawal Scale; DSM: Diagnostic and Statistical Manual of Mental Disorders; EHR: electronic health record; SL/PO: sublingual/by mouth.

These suggestions led to a complete redesign of the CDS in prototype 3 (Figure 3). To improve clarity and consolidate steps, this version included all treatment options in a single table on 1 screen with a row for each treatment option. User feedback for this version focused on optimizing the design by changing fonts, reducing the amount of text, and labeling treatment options and the decision support tools appropriately.

**Figure 4:**
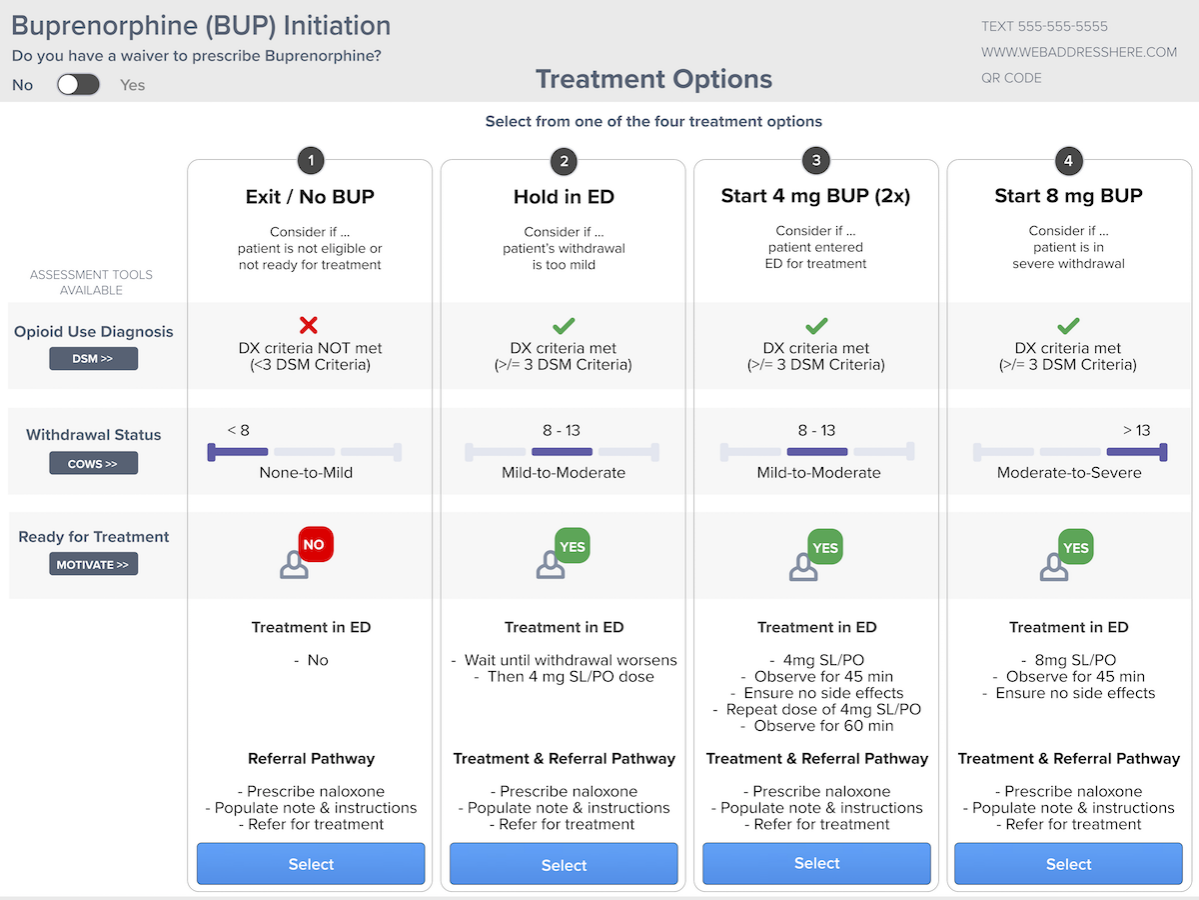
Fourth prototype user interface with care pathways to columns. BUP: buprenorphine; COWS: Clinical Opioid Withdrawal Scale; DSM: Diagnostic and Statistical Manual of Mental Disorders; SL/PO: sublingual/by mouth.

This feedback informed the design of prototype 4 (Figure 4) in which treatment pathways were presented in columns (rather than rows) with 1-click treatment pathway selection at the bottom of each column. Buttons in the far left column provided access to modules for OUD diagnosis, withdrawal assessment, and motivation and assessment of patient readiness for treatment. Feedback for this version was mostly positive, with minor navigation concerns about where to start the tool and how to activate the decision support.

### Phase 4 (Final Prototype Testing)

**Figure 5:**
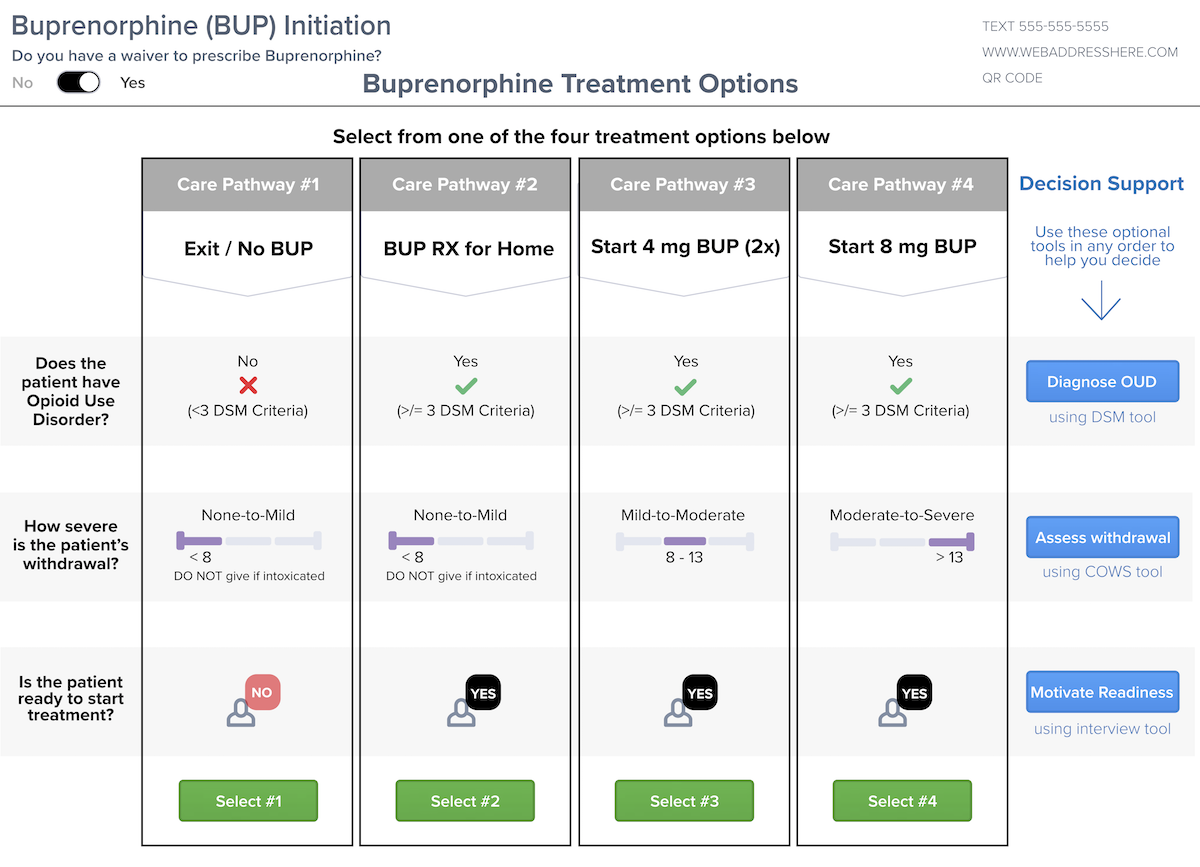
Final prototype user interface with decision support moved to the right column. BUP: buprenorphine; COWS: Clinical Opioid Withdrawal Scale; DSM: Diagnostic and Statistical Manual of Mental Disorder; OUD: opioid use disorder.

**Figure 6:**
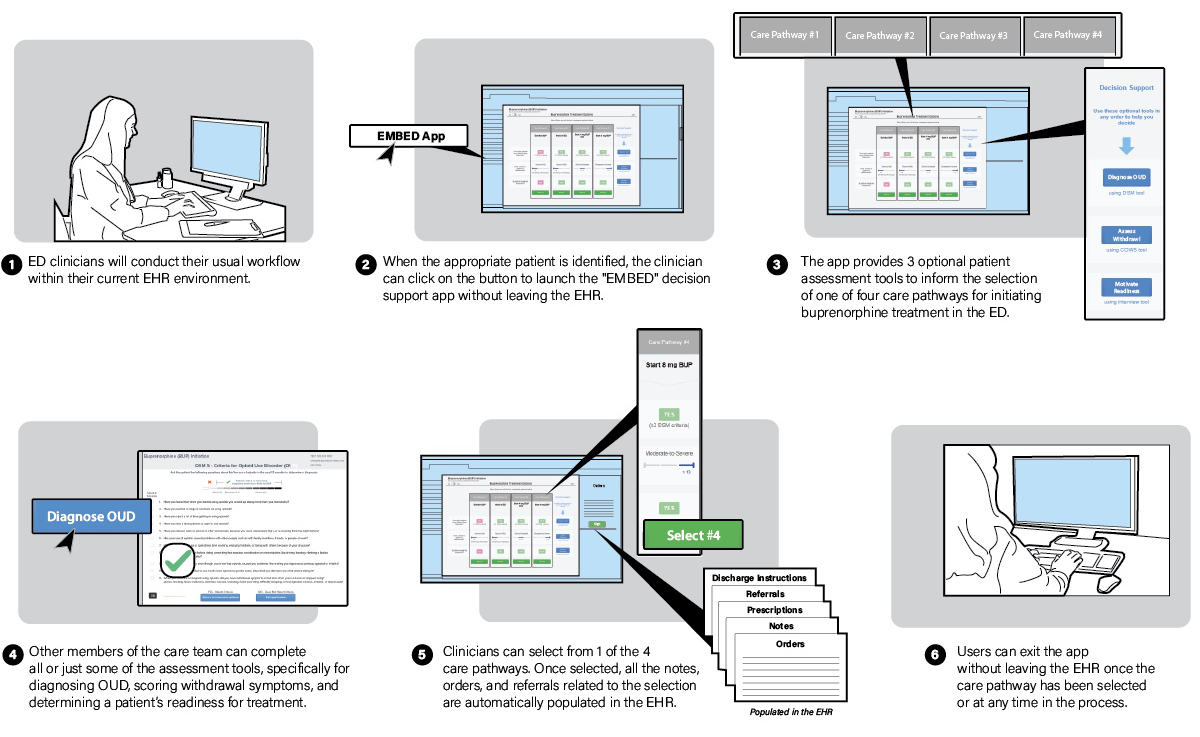
Final information technology workflow for user-centered clinical decision support intervention based on formative evaluation. ED: emergency department; EMBED: EMergency department-initiated Buprenorphine for opioid usE Disorder; EHR: electronic health record; OUD: opioid use disorder.

The final prototype design goal was an intuitive, simple layout offering flexibility for direct treatment or user-initiated decision support. In response to navigation concerns, nonessential text was removed, and decision support was presented with blue buttons in the far right column, following the horizontal path for the DSM, COWS, and motivational interview (Figure 5). Although feedback was generally positive for the simplified layout, users suggested that the direct care pathways needed clear delineation. These concerns were addressed in a final design change to outline each treatment column. During final testing, multiple users initially attempted to click in the middle of the main screen to select a care pathway, so care pathway activation buttons were changed to green to indicate the start of treatment. All participants at this stage thought that the system was easy to learn without training and reasonable for use in their routine emergency care practice. Figure 6 summarizes the needs assessment and general workflow of our intervention based on all phases of formative evaluation.

## Discussion

### Principal Findings

We describe the iterative user-centered design process to develop a CDS for ED-initiated BUP. Across 4 phases with 5 major revisions and continuous iteration, we identified user needs for a flexible tool to support members of the care team who could be either experienced users or those new to ED-initiated BUP. Interactive feedback sessions identified key themes throughout the refinement process, including issues of navigation, overall design recommendations, considerations for workflow integration, and questions regarding the treatment protocol. Throughout the design process, how and where to activate decision support represented a key challenge. Early prototype versions provided *step-by-step* guidance through existing forms and processes. Requests for a more efficient and flexible tool resulted in an easy-to-read layout with options for direct clinical care activation or user-activated decision support. The challenge of simultaneously providing both a direct care pathway and flexible decision support led to a design with multiple navigation options. Adoption of key design principles included minimizing unnecessary text, utilizing a standard form and colors for buttons, and layout of information in meaningful pathways. We selected a final design once all user-identified concerns had been addressed and user feedback indicated the tool was meeting their anticipated needs.

### Strengths and Meaning of the Study

This study supports the use of user-centered design. Through both formal and informal feedback, we captured user needs and input that would not be captured in traditional CDS design processes that lack a needs assessment or formative evaluation. Expert review of content ensured accuracy, whereas feedback from users with varying levels of experience highlighted the need for flexible support. Comments from expert users emphasized the need for an option to directly launch the desired care pathway. Although a direct care pathway provides flexibility for expert clinicians, novice clinicians emphasized the need for more structured decision support and clarity in the diagnostic and treatment processes. Specifically, less experienced users welcomed the detailed criteria for OUD diagnosis and withdrawal assessment, instructions for conducting a motivational interview, and clarity regarding BUP dosing. This contrast in user needs presented a design challenge that highlights the importance of sampling participants across the range of user experience levels with the protocol supported by the CDS. A deliberate sampling of participants from earlier iteration sessions as well as new participants provided confirmatory feedback on how recommended changes were incorporated into the design. Finally, design relied on both user comments and existing standards for layout and use of specific design features such as color.

### Limitations

As the CDS supports a treatment pathway, the underlying workflow driving development was identified as clinician workflow. As such, clinicians were the primary users studied. Less focus on other members of the care team could represent a limitation—in particular, if the user population is broadened in implementation at the site where the design process was conducted or at other sites using the tool in the future. A number of users suggested a role for other nonclinician staff in identifying OUD patients in the ED. In particular, multiple clinicians mentioned that the COWS could be completed by a nurse. Therefore, the tool is designed with resources that can be used by or distributed to other members of the care team (eg, nurse, medical student, and addiction counselor). In this way, a nonclinician could still complete the diagnostic or withdrawal assessment, and the clinician could incorporate this assessment into their final care pathway selection.

Given the urgency of the opioid epidemic, we made a conscious decision to take a pragmatic approach to the design and formative evaluation of our intervention. Developing the CDS through a pragmatic approach instead of a traditional academic approach allowed for the rapid inclusion of user feedback in a shorter time frame [29]. We recognize that limitations to this approach exist, including the potential for additional data that could be captured in a deeper, more rigorous data analysis typical of the academic approach. With a traditional academic approach, data saturation anticipates capturing 100% of user feedback themes, whereas this pragmatic approach to development relies on capturing 80% of critical issues [36]. However, we were willing to accept this trade-off to achieve the aim of the subsequent trial to accelerate getting this life-saving treatment into routine emergency care.

This work represents the initial phase of a larger project for the development, implementation, and testing of the effectiveness of the CDS developed here. Design and user feedback sessions were conducted at a single site, though implementation will include multiple sites and could potentially interface with other vendors’ EHRs. Having a limited group of users engaged in design is practical and not unique to our work. However, we recognize that this introduces the potential for design features supporting local norms and processes that may not be generalizable. To mitigate this potential limitation, we sought feedback throughout the design process from external collaborators as well as guidance from a subject matter expert on ED management of substance use disorder.

### Comparison With Prior Work

Given the devastating toll of the opioid epidemic, this user-centered CDS was developed to give clinicians the tools necessary to engage more people suffering from OUD in effective treatment at a time when they may be particularly open to it [8-10]. However, this intervention may be challenging to disseminate for several reasons: (1) it implements a multistep practice that is not familiar to clinicians; (2) ED clinicians are unlikely to see immediate effects of their efforts; (3) the targeted patient population is often perceived to be difficult to work with; and (4) the legal status of BUP for OUD is complicated, requiring a special waiver to prescribe for home use, but no waiver required if the treatment is administered onsite for no more than 72 hours [37,38].

Given these challenges to adoption, we perceived an opportunity to increase the likelihood of success by employing user-centered design to create the EMBED CDS intervention. Emerging literature supports this approach [39-42]. For example, Thursky and Mahemoff have incorporated participatory design methods to create antibiotic CDS for physicians in intensive care units [39]. Kilsdonk et al have employed user-centered decision support to create a tool that improved the speed and accuracy of clinician’s identification of appropriate screening procedures for childhood cancer survivors, relative to the use of a paper guideline [40]. Plaisance et al have used a process similar to our study to design a CDS for cardiopulmonary resuscitation in the intensive care unit [42]. We have also previously employed a user-centered design in developing CDS for patients with head injury in the ED [41].

Notably, these studies have shown that including user feedback in the design phase leads to greater effectiveness and efficiency and, ultimately, to a sense of physician ownership of the CDS, which increases its immediate uptake and continued use. They have also highlighted rapid-cycle prototyping with user engagement throughout a design process [42]. Similar to previous reports in this area, we found that different groups of users expressed different needs for the tool. We approached this challenge through a design approach that balanced the goals, priorities, and information processing needs of both novice and expert users. This resulted in a tool that could support multiple types of users and their preferred workflows. We demonstrate how a single tool can be designed with the flexibility to meet multiple users’ work processes and information processing needs. Designing for the human requires an understanding of workflows, information needs, priorities, and preferences; user-centered design captures this through user engagement across the design and development life cycle [26-28].

### Conclusions

This work describes the design and formative evaluation of a user-centered CDS for ED-initiated BUP. We add to the expanding literature on the design of user-centered CDS tools by describing the process and challenges of designing a flexible tool that supports both novice and expert clinicians in identifying appropriate patients and appropriate care pathways. Future work will include summative usability evaluation and pilot testing of the intervention to further optimize the tool for wide-scale implementation within existing ED workflows in a large pragmatic clinical trial across multiple health care systems. The aim of this subsequent pragmatic trial is to increase adoption of ED-initiated BUP for people suffering from OUD, thereby decreasing morbidity and mortality associated with opioid addiction. Users will also inform pilot implementation in a series of focus groups. Although early engagement of users supports the design process, we anticipate continued support of potential users will be equally important across the project life cycle.

## Appendix

Multimedia Appendix 1 User-centered design script.

Multimedia Appendix 2 Design iterations, feedback, and solutions.

## Abbreviations

BPA: best practice alert
BUP: buprenorphine
CDS: clinical decision support
COWS: Clinical Opioid Withdrawal Scale
DSM: Diagnostic and Statistical Manual of Mental Disorders
ED: emergency department
EMBED: EMergency department-initiated Buprenorphine for opioid usE Disorder
OUD: opioid use disorder
