# Supplementary material for: Computerized Clinical Decision Support System for Emergency Department–Initiated Buprenorphine for Opioid Use Disorder: User-Centered Design
Source: JMIR Hum Factors. 2019 Feb 27;6(1):e13121. doi: 10.2196/13121 (PMC6414819; doi:10.2196/13121)
Supplement: Multimedia Appendix 1 [file humanfactors_v6i1e13121_app1.pdf]

EMBED User Centered Design Script

Thank you for participating in this user centered design session. This session will focus on the design of a clinical decision support system for ED-initiated Buprenorphine/naloxone (BUP). We are currently in [early prototypes] of this tool and are gathering feedback from future users. The session will take approximately 5-10 minutes of your time. First, I will present you with a case scenario of a patient presenting in the ED during your shift. I will then present you with a series of slides representing the decision support tool. As you review each slide I will ask you to talk aloud as you have initial reactions to the information presented and how you would respond to each slide. I will also ask for each interaction with the tool (i.e. checking a box) what you would expect from the system. Please feel free to ask questions at any time.

Are you ready to begin?

Case Presentation:

You are in the middle of a busy B-side shift in the York Street ED. You just transferred a critically ill septic patient to the A-side. During that process, you notice that there are now 4 patients to be seen in your zone. Before picking up a new case, your attending asks you to reassess Mr. Coleman who has been waiting to be discharged for a few hours after an initial presentation for heroin overdose and narcan revival by EMS.

The patient is 26-year-old single male who was transported to the ED from his home by ambulance following a 911 call by a friend and naloxone administration by EMS. He has no known medical history and no allergies to medication, etc. He reports resuming use of heroin in the last 2 weeks after completing a 28-day inpatient/residential treatment program. He indicates that he is using 4 bags of heroin per day, which is up from 1-2 bags/day in his first week post-discharge from the rehab. He is concerned that he will quickly go back to his “bundle” (10 bag)/day pattern, but states adamantly that he does not want to go back to rehab.

You enter to reassess Mr Coleman.

How would you recognize this patient is a potential candidate for Buprenorphine? (*BPA)*

Show first screen – Buprenorphine Initiation Process: Plan

Prompt: Talk me through your reactions and how you would use this tool…

When user says “click start” show Diagnosis screen

Prompt: Describe what you expect to happen as you enter your responses?

Prompt at third item selected: Once you have selected the three items the patient will be identified as moderate to severe and you can move to the next screen.

Show Withdrawal Scale Screen

Prompt: Talk me through how you would use this screen

Once user has described ratings and states "click next" show Readiness for Treatment

Once user has described rating scale show Dosing screen

Prompt: What would you do with the information on this screen?

Prompt: Is there any additional information you need?

Once user states "click next" show Referral Screen

Prompt: What would you do with the information on this screen?

Prompt: Is there any additional information you need?

Debrief questions:

What is your overall impression of this tool?

What would make the tool easier for you to use in practice?

What would increase the likelihood of you incorporating this tool in your practice?

Do you have any additional thoughts or suggestions you would like to share?
