# Supplementary material for: Computerized Clinical Decision Support System for Emergency Department–Initiated Buprenorphine for Opioid Use Disorder: User-Centered Design
Source: JMIR Hum Factors. 2019 Feb 27;6(1):e13121. doi: 10.2196/13121 (PMC6414819; doi:10.2196/13121)
Supplement: Multimedia Appendix 2 [file humanfactors_v6i1e13121_app2.pdf]

Design iterations, feedback, and solutions.

Primary design characteristics/considerations	Feedback	Design considerations/approach
**Initial Prototype Design—BPA[a]**		
BPA; Yes/No/Unclear checklist to activate CDS[b] or start treatment protocol	—	—
Navigation: Accessing Decision Support	To alert MD[c] of clinical decision support resource use a BPA	Used prior BPA as a format for ways to link to identifying right patient, starting CDS, or skipping it
	Need to have a way of skipping portions of the process, for example, if OUD[d] or withdrawal is obvious, jump to treatment	Yes, No, Unclear check boxes to skip portion of the process if decision support is not needed
Treatment Process	—	Incorporate DSM[e] checklist: Designed a "select all" dropdown menu to count and calculate symptoms
	—	Incorporate COWS[f] checklist: Designed both a Likert scale and a dropdown menu approach
Workflow Integration	Use workflow diagram to guide dosage process	Created step-by-step guide to accommodate different dosage amounts and un/waivered physicians
	Needs to be completed in 2-5 min	Flexible navigation to skip portion of process if CDS not needed
**Prototype Iteration 2: User-activated decision support**		
Initial page with left column initiation and right panel for	—	Prototype 1, the format of a CDS that included the

support: Checklist for patients meeting criteria (DSM/COWS/Readiness); Click i for decision support access		collection of checklists, decision support, and forms in their fullest format, was just too much information to work through and thus required a significant refinement to focus and streamline the material in a simplified UI .... and so, a second version of the UI was started with the goal of reducing information and to reside within Epic
Navigation	Need a navigation bar after the home screen with the ability to navigate from one module to the next in the desired order: Diagnosis>Withdrawal Scale>Readiness for Treatment>Dosing>Referral	Navigation design change to single click and decision support access specific to DSM, COWS, and interview
	How to enter the tool is not clear	Added i buttons to allow independent access to decision support for each DSM, COWS, and interview
	Check boxes confusing - I expect tool to help me answer these. Do I check boxes to begin?	Redesign to remove check boxes for treatment activation
Treatment Process	Liked the general concept of standardizing COWS scale, but warned against using the None>Mild>Moderate>Moderately Severe>Severe Likert scale. There are 4 categories, not 5 for	Replaced sliding scale with clickable descriptive scale

	the majority of the questions	
	#3 is confusing. Consider returning to ED in 2 days...Do I write prescription or not?	Revised wording
	Not clear what to prescribe for patient leaving. “What do they take for post discharge?”	Revised wording
	“Do you have to meet all three check boxes on left to treat?”	Redesign to offer visual guidance on criteria for each treatment pathway
Workflow Integration	Treatment Screen: please add a link to print, email, text the 1-page algorithm here in case they leave the computer and want to reference it away from the EHR[g]	Added text number for access outside of tool
	Unless offering more than BUP[h] plan, intervention may be better off as a Procedure Note workflow	Consideration for implementation
Design Recommendation	Treatment screen: remove the timers, I do not think we will be able to build in that functionality. We can still give the clinician a recommendation though.	Included time recommendations in treatment instructions
	Consolidate to fewer screens: Keep the Plan questions as a sidebar showing progress (and navigation)	Maybe this just becomes a checklist? Where you order and refer to treatment if you meet DSM and COWS criteria. But either way, it does bring up that we may have too many redundancies in the navigation bar at top

		and bottom without the user having a continuous visible status for their progress in moving toward treatment
	Consolidate to fewer screens: Only one more tab for treatment and referral	If you are treating, you should be referring. So, tab could be removed and what lives in the Referral tab now could prepopulate fax/email/page to MAT[i] referral center
	Like the idea that at a threshold you do not have to answer any more questions. Do not want to have to answer all questions	Move forward with design that informs user when criteria are met
	Recommend just the quick interview. Additional information clutters page and takes too much time	Retain short interview prompts on page with i button for full interview details
	Prescription recommendation/instructions—would love to have that populate (or copy/paste) for discharge instructions	Populate Note, Orders, Rx[j], and Discharge instructions based on care pathway
**Prototype Iteration 3: Single click for Direct Care Pathways (rows)**		
Four direct pathways to activate treatment presented in rows; Click i at top of column for decision support access	—	At this point, the prior versions of the prototype had too much text to work through and too many steps to navigate. Still a feeling that this was something that doctors would find too cumbersome. So, basically

		scrapped the prior direction altogether and tried a new format that aimed to organize the data into a table with the columns outlining the treatment options ... with the goal for the user to see all options at once, and then select the best choice
Navigation	Can we get to just one click?	Move to Treatment pathways with single click activation on front page
	Need to structure the options	Launching COWS, DSM, BNI[k], were identified with "i" icon
Treatment Protocol	Support waivered and nonwaivered options	Toggle button for waivered/nonwaivered
Design Recommendation	Need to be limited to one page if possible	Columns with Exit, Hold, and Start BUP options
	Too much text for COWS	Working with experts we limited text on COWS
**Prototype Iteration 4: Single click for Direct Care Pathways (columns)**		
Four direct pathways to activate treatment presented in columns; Gray buttons to access decision support in left column	—	Evolved the format to be more legible in a "Rows" structure, maintaining the "toggle" for providers, emphasizing 4 "buttons" at the end of the rows for treatment decisions, and adding more graphical depictions for DSM, COWS, and BNI, for example, check

		marks, Yes/No, scales. Links to decision support tools, such as DSM, COWS, and Interviews, were placed at the top of the columns
Navigation	Lucky guess on entering CDS	Added text to highlight decision support, moved buttons to right column, and colored used to indicate information
	New screen seems straightforward, except I am looking for/expecting a "do you want to do this?" prompt	Added text to highlight decision support
	Assessment/Decision Tools not obvious—needs “START HERE”	Added text to highlight decision support
	Information on interview needs to be sooner—not interested in going through all these steps if patient is not interested	Independent navigation for decision support for each DSM, COWS, and motivational interview. Added text to indicate these were optional and could be completed in any order
Treatment Protocol	Is there a cutoff score on the motivational interview for patient being ready for treatment? I need to select, but do not see a score	Included descriptive text on interview support page to guide use of interview response
	If not in withdrawal, do we have to wait? Need clearer statement “Do not give if intoxicated”	Added wording “Do not give if intoxicated” to front page
Design Recommendation	“Will COWS stop once you get too severe?” (Similar to DSM?)	Included progress bar to indicate when Moderate-to-Severe is reached

	Entry screen needs less/simpler information	Removed the text in each care pathway below Ready for treatment as that text is now on the Thank You screen. That is, delete “Treatment in ED” and “Treatment & Referral Pathway”—all of those details will pop up when you click “Select” and move to the treatment screen
	The vertical boxes 1-2-3-4 currently seem like where I should start so I did not pay attention to the other parts of the screen	Changed numbers to “Care Pathway 1,2,3,4”
**Final Design: Decision support moved to right column**		
Blue buttons in right-hand column to access decision support; Removed redundant text	—	Observations and feedback identified continued issues with navigation. Design changed and moved buttons for CDS to right column at the end of each treatment decision row (DSM/COWS/interview). Refined use of color and button style.
Navigation	Appeared to access decision support naturally—when questioned responded if he was not sure if that was design or because he had seen a previous iteration	—
	Buttons of right sidebar make	—

	sense	
	(3 faculty summary) They all clicked on the middle of the main screen thinking it would launch the care pathway (though 1 thought it would launch the CDS for that row). All 3 felt that the care pathways should be more discrete columns to help convey this. They all felt that once they had used it once, this would not be an issue though. The user who had seen it before wondered if changing the button color would make this more clear but focused more on the column issue.	Incorporated column borders from earlier design and made care pathway buttons green. Retained blue decision support buttons in the right-hand column
Design Recommendation	Not clear that note on Thank You page is going into chart	—
	Like the design revisions - it is cleaner	—

—: not all cells in this table are completed as not all design feedback led to a design change and sometimes design changes were made based on multiple pieces of feedback per the design team's assessment.

[a]BPA: best practice alert.

[b]CDS: clinical decision support.

[c]MD: doctor.

[d]OUD: opioid use disorder.

[e]DSM: Diagnostic and Statistical Manual of Mental Disorders.

[f]COWS: Clinical Opioid Withdrawal Scale.

[g]EHR: electronic health record.

[h]BUP: buprenorphine.

[i]MAT: medication for addiction treatment.

[j]Rx: prescription.

[k]BNI: brief negotiated interview
